# Effect of Metal Oxide Nanoparticles on Microbial Community Structure and Function in Two Different Soil Types

**DOI:** 10.1371/journal.pone.0084441

**Published:** 2013-12-13

**Authors:** Sammy Frenk, Tal Ben-Moshe, Ishai Dror, Brian Berkowitz, Dror Minz

**Affiliations:** 1 Institute for Soil, Water and Environmental Sciences, Agricultural Research Organization, Bet-Dagan, Israel; 2 Department of Environmental Sciences and Energy Research, Weizmann Institute of Science, Rehovot, Israel; 3 Robert H. Smith Faculty of Agriculture, Food and Environment, The Hebrew University of Jerusalem, Rehovot, Israel; Wageningen University and Research Centre, Netherlands

## Abstract

Increased availability of nanoparticle-based products will, inevitably, expose the environment to these materials. Engineered nanoparticles (ENPs) may thus find their way into the soil environment *via* wastewater, dumpsters and other anthropogenic sources; metallic oxide nanoparticles comprise one group of ENPs that could potentially be hazardous for the environment. Because the soil bacterial community is a major service provider for the ecosystem and humankind, it is critical to study the effects of ENP exposure on soil bacteria. These effects were evaluated by measuring bacterial community activity, composition and size following exposure to copper oxide (CuO) and magnetite (Fe_3_O_4_) nanosized (<50 nm) particles. Two different soil types were examined: a sandy loam (Bet-Dagan) and a sandy clay loam (Yatir), under two ENP concentrations (1%, 0.1%). Results indicate that the bacterial community in Bet-Dagan soil was more susceptible to change due to exposure to these ENPs, relative to Yatir soil. More specifically, CuO had a strong effect on bacterial hydrolytic activity, oxidative potential, community composition and size in Bet-Dagan soil. Few effects were noted in the Yatir soil, although 1% CuO exposure did cause a significant decreased oxidative potential and changes to community composition. Fe_3_O_4_ changed the hydrolytic activity and bacterial community composition in Bet-Dagan soil but did not affect the Yatir soil bacterial community. Furthermore, in Bet-Dagan soil, abundance of bacteria annotated to OTUs from the Bacilli class decreased after addition of 0.1% CuO but increased with 1% CuO, while in Yatir soil their abundance was reduced with 1% CuO. Other important soil bacterial groups, including *Rhizobiales* and *Sphingobacteriaceae*, were negatively affected by CuO addition to soil. These results indicate that both ENPs are potentially harmful to soil environments. Furthermore, it is suggested that the clay fraction and organic matter in different soils interact with the ENPs and reduce their toxicity.

## Introduction

Materials at the nanometer scale are not new, having been reported to be found naturally in various environments, including volcanic dust [1], oceans [2], fresh water [3] and soils [4,5]. On the other hand, anthropogenic engineered nanoparticles (ENPs) have appeared relatively recently, with their manufacturing and use becoming widespread only during the last decade [6]. ENPs are employed in the cosmetics industry [7], antimicrobial paints [8], electronic devices, and textiles [9]. ENPs are often designed to be extremely reactive, and they have characteristics, unlike some of their natural counterparts, that may be harmful to different life forms including microorganisms and animals. The presence of anthropogenic ENPs in the environment, particularly TiO_2_, has already been reported in streams [8] and wastewater treatment plants [10]. Indeed, the increase of new ENP-based products promises a steady increase in ENP production, availability and ultimately discharge to the environment [11]. 

ENPs can be classified by size, structure, toxicity and obviously by their chemical composition. Metal oxide ENPs (MO-ENPs), which include CuO, TiO_2_, ZnO, CuZnFe_2_O_4_, Fe_3_O_4_, and Fe_2_O_3_ among others, are used extensively in a variety of applications, despite the fact that few studies have examined their potentially hazardous effects to the environment [11,12]. MO-ENPs affect the environment differently than their bulk size or dissolved ion counterparts, because of their high surface to volume ratio [13,14]; surface characteristics such as charge and reactivity are intensified in MO-ENPs, which therefore make them potentially more dangerous to organisms. Many other variables are involved in determining MO-ENP toxicity, including size, oxidative state, exposure time, particle concentration and the target organism [12–15]. Moreover, when evaluating the toxicity of MO-ENPs to organisms living in a habitat such as soil, other variables must be taken into account, including soil type, soil water content, soil organic matter and mineral composition [12–17]. 

Soil microbial communities are responsible for many of the biogeochemical processes on Earth, such as nutrient mineralization, nitrogen cycling and organic carbon degradation [18,19]. Therefore, many ecosystem services, including supply of clean groundwater, waste degradation and agricultural production, are dependent on the well-being of the soil microbial community. Changes in microbial activity and community composition can result from changes in availability of nutrients and organic carbon, anthropogenic activity and introduction of contaminants such as MO-ENPs. Identifying and characterizing affected groups of microbes and quantifying the productivity of the community are essential for the characterization of MO-ENP effects, designing toxicity detection methods, and as a step toward defining improved bioremediation practices. 

Most of the studies on environmental impacts of MO-ENP contamination have focused on determination of lethality to specific organisms, including fish [20,21], crustaceans [22–24] and the bacteria *Vibrio fischeri, Bacillus subtilis* and *Escherichia coli* [23,25]. Recently, a disruptive effect of 100 ppm CuO ENPs was reported for decomposition of plant litter by the microbial community in streams [26]. In concurrence with these findings, disruptive effects to soil bacterial communities were detected by ZnO and TiO_2_ in a dose-dependent manner [27]. A negative effect was also detected in arctic soil microbial communities by the addition of 0.066% (w/w) silver (Ag) ENPs, but surprisingly, not for copper or silica [28]. Similarly, Shah and Belozerova [29] found no effects of a variety of metallic ENPs on a soil microbial community in the presence of relatively high nutrient levels. Collins et al. [30] described the effect of Cu and ZnO ENPs on different soil horizons over a 160 day period and showed that different bacterial orders are affected differently by these contaminants. For example, *Flavobacteriales* and *Sphingomonadales* were affected negatively by ENPs, whereas *Rhizobiales* were much more resilient to both contaminants. Ge et al. [27] found similar responses of *Sphingomonadales* and *Rhizobiales* to ZnO to those of Collins et al. [30]. Overall, it appears that there are contradictory findings regarding the environmental toxicity of MO-ENPs at the community level. In contrast, some consistent trends were found regarding the effect these materials have on specific bacterial groups. Further research is needed to understand the effect of MO-ENPs on the microbial communities under different conditions, especially for different soil types.

In this study, we assessed soil microbial activity and composition of soil bacterial communities following exposure to two MO-ENPs, copper oxide (CuO) and magnetite (Fe_3_O_4_), in two soil types (sandy loam and sandy clay loam). We hypothesized that soil type is a deterministic factor dictating the vulnerability of soil organisms to MO-ENP pollutants. The selected soil types are representative of two different soil textures and characteristics, which are common in Israel and other regions in the world. Furthermore, we explored the effect of concentration of these MO-ENPs by comparing two different contaminant doses, 0.1% and 1% w/w. The lower concentration is in the range often tested in different habitats and with different MO-ENPs [28,29,31,32]. The higher concentration, on the other hand, is in a range estimated to be found in cases of spills from industrial sources. We hypothesized that a 0.1% dose would cause a slight change in the community function and structure, while a 1% dose would affect these parameters more significantly. We further attempted to identify bacterial populations with specific sensitivity or, on the other hand, robustness in the presence of the contaminants. 

## Materials and Methods

### Experimental design

This study was conducted at the Agricultural Research Organization, Bet-Dagan, Israel. Two types of soil, sandy loam from Bet-Dagan, Israel (pH 7.7, clay: 16.2%, silt: 6.3%, sand: 77.5% collected from a site located at 31°59'N34°49'E) and sandy clay loam from Yatir, Israel (pH 7.5; clay 36.9%; silt 14.8%; sand 48.3%, located at 31°21'N35°1'E), were chosen for the experiments. The authorization to collect the samples from Bet-Dagan soil was given by the Agricultural Research Organization and for the Yatir soil from Keren Kayemeth LeIsrael – Jewish National Fund. The samples were taken from the upper layer (0-10 cm) of the soil and sieved through a 60 mesh (approximately 250 μm pore size) strainer. Nanoparticles at doses of 0.1% or 1% (w/w_air dried_) of CuO or Fe_3_O_4_ (Aldrich, <50 nm) were used. The experiments were performed with 80 g of air dried (water content of 0.67% and 1.1%, for Bet-Dagan and Yatir soil, respectively) soil in 100 mL sterilized glass bottles. The contaminants were mixed overnight by mechanical shaking (MRC shaker SOH 3030) with dry and sieved soil until achieving a homogeneous texture. Deionized water was added to control and contaminated soil samples to reach a final water content of 30% (w/w), which were then incubated at 30 °C for 48 h as described previously [17]. All treatments, including the unexposed controls, were replicated 5 times and placed in an incubator. The samples were fully characterized in terms of their chemical composition (mineral concentrations, organic matter and pH) and physical properties (porosity, hydraulic conductivity, FTIR and fluorescence analysis, scanning electron microscopy (SEM), energy dispersive X-ray spectroscopy (EDS) and the amount of nanoparticles deposited on the soil), as previously described [17]. 

A control experiment was set up for the evaluation of non-biological CO_2_ emission and interference with fluorescein di-acetate (FDA) and dehydrogenase (DEH) enzymatic assays. This included a sterilization procedure and was done for the Yatir soil only, as results of the main experiment showed Yatir soil to be robust to the effect of ENPs. This experiment included (i) uncontaminated, unsterilized control, (ii) uncontaminated, sterilized control, (iii) unsterilized samples contaminated with 1% of either ENP, and (iv) sterilized samples contaminated with 1% of either ENP, all with three repetitions. The sterilized controls of uncontaminated and 1% w/w ENP-exposed soils were autoclaved at 121 °C for 55 min three consecutive times, with a 24 h period between each autoclave treatment. In all samples the different ENPs were added after the soils were sterilized. The incubation procedure in this control experiment was identical to the main experiment. At the end of the incubation, in both experiments, a portion (65-90 g wet weight) of each sample was oven dried at 105 °C for 24 h to calculate water loss during the experiment and to normalize the different assays (enzymatic activities and 16S rRNA gene copies) to dry weight. 

### Enzymatic assays

Respiration rates were measured for three of the five replicates using an acid-titration technique [33]. Briefly, a sample of 40 g of soil in a closed jar was incubated for 24 h at 30 °C with 2 mL of 1 N NaOH trap, followed by acid titration to quantify the CO_2_ evolution. Soil oxidative potential was estimated by measuring DEH activity [34] using 2,3,5-triphenyltetrazolium chloride as substrate. The resulting formazan was measured by spectrophotometry at 494 nm with a DMS100 UV visible spectrophotometer (Varian Inc., San Fernando, USA). Hydrolytic activity was measured by FDA hydrolysis assay as described by Schmidt and Belser [35]. The amount of hydrolyzed FDA was measured by a spectrophotometer at 494 nm. The results of these measurements are described as percentage from the average measured activity in the control samples, although the statistical analyses were performed on the raw data. 

### DNA extraction

DNA was extracted from 0.5 g soil samples *via* bead beating (Fast Prep FP 120, Bio101; Savant Instruments Inc., Holbrook, NY) in extraction buffer [100 mM Tris HCl, pH 8.0; 100 mM potassium phosphate buffer pH 8.0; 1% cetyltrimethylammonium bromide (CTAB)] [36]. The crude extracts were mixed with KCl to a final concentration of 0.5 M, incubated for 5 min, and centrifuged. The nucleic acids were then precipitated by centrifugation using an equal volume of isopropanol. The pellet was washed with ice-cooled 80% ethanol and centrifuged. The pellet was eluted using 10 mM Tris-EDTA (TE) at pH 8.0 (Amresco Inc., Solon, OH). DNA present in the solution was then bound to “glassmilk” 0.5 to 10 μm silica particles (Sigma-Aldrich, St. Louis) with NaI, as described by Reference 37. The “glassmilk” was transferred to a Spin-X^®^ 0.22 μm filter tube (Corning, NY) and washed with an ethanol-based wash buffer solution [37]. DNA attached to the silica was eluted with TE into a sterile tube and stored at -20° C prior to use. Prior to any downstream applications, the DNA concentration and purity was measured by optical density using a NanoDrop ND1000 spectrophotometer (NanoDrop Technologies, Wilmington, DE, USA) to account for any treatment-related bias in extraction yield.

### 454 high throughput sequencing and quantitative PCR

Sequencing using 454 tag-encoded FLX 16S rRNA gene amplicon pyrosequencing (bTEFAP) was performed at the Research and Testing Laboratories (Lubbock, TX, USA). PCR targeting the 16S rRNA gene was done using the 16S rRNA gene bacterial primers, 530F, GTG CCA GCM GCN GCG G and 1100R, GGG TTN CGN TCG TTG as described previously [38]. Two replicates were sequenced for all of the treatments except for treatments of 1% CuO and 0.1% Fe_3_O_4_ where three replicates were performed. The data were submitted to the Sequence Read Archive public repository under the study accession ERP001973. In addition, the evaluation of total bacteria in soil samples was performed by quantitative PCR with Absolute Blue SYBR green ROX mix (Thermo Fisher Scientific, Surrey, United Kingdom), as described previously [39]. We used a general bacterial primer pair targeted at the 16S rRNA gene, Eub515F, GTG CCA GCM GCC GCG GTA A and 907R, CCG TCA ATT CMT TTG AGT TT [40]. Four replicates were done in this method including three pseudo-replicates for each biological sample. A plasmid standard containing the 16s rRNA gene target region was generated and its concentration was determined with the Nano-Drop ND1000 spectrophotometer. Real-time PCR assays were conducted in polypropylene 96-well plates in a Mx3000P QPCR system (Stratagene, La Jolla, CA) and the results were normalized to soil weight.

### Sequence preparation and analysis

Sequences preparation, including trimming of tags and primers, quality assurance and chimeric sequence removal for 454 data analysis were done using MOTHUR 1.23.1 [41] software under the guidelines of the standard operation procedure [42]. The sequences were first trimmed of primer and tag sequences, and then selected by quality (using “average window”, average quality>30, window size=50 bp) and length (length>200 bp). The sequences were aligned using a reference database (Silva database) and then filtered to 624 aligned characters. Chimera detection was performed with the “chimera.uchime” command, using the most common sequences of each soil sample as references. The remaining sequences were clustered to unique (0.999% similarity) and the “pre.cluster” function was applied (1 mismatch for each 100 bp). A distance matrix was calculated and the sequences were clustered into operational taxonomic units (OTUs) on a 97% sequence similarity basis. As the sequence number was different among samples, a “sub.sample” command was used and 752 sequences were randomly selected from each sample, number that equals to that of sequences in the sample with the lowest yield. 

### Statistical analyses

The community composition was described using two methods. First, denaturing gradient gel electrophoresis (DGGE) fingerprinting and its interpretation (see supplementary material) enable a simple but robust visualization of the bacterial community composition. The same samples were further analyzed using high-throughput sequencing of 16S rRNA gene fragments. The sequencing data enable both recognition of the bacterial genera affected by MO-ENP application and use of statistical hypothesis testing methods to confirm these changes. The results of the two methods are reported and discussed here concurrently, but the statistics are relevant only to the sequencing data. OTU analysis included calculation of richness, sampling coverage and between-group differences based on the weighted UniFrac pairwise distance matrix. The observed richness was calculated by the number of OTUs detected in a sample and the coverage was calculated using Good’s coverage estimator [43]. The similarities between the bacterial communities were ordinated using PCoA plots. These plots were based on weighted UniFrac pairwise distance matrix [44] of all samples and separately for each soil type, and calculated from a neighbor joining tree of all sequences. Hypothesis testing statistical analysis of molecular variance (AMOVA) [41,42,45] was performed on the samples in the weighted UniFrac pairwise distance matrix grouped by treatments (2 or 3 replicates) and results were corrected using the Bonfferoni correction method for multiple comparisons. Phylogenetic classification was done using the Silva taxonomy reference file available on the MOTHUR website. Sequences were then clustered to class level phylogeny. Finally, all samples were compared using the “Metastats” function which determines whether which of the OTUs are differentially represented (in terms of relative abundance) between the treatments in a significant manner (p<0.05) [46] within each soil type.

Two-way analysis of variance (ANOVA) was applied to test for the effects of MO-ENP concentration and soil type on bacterial activity, abundance and OTU richness. Data were analyzed for each MO-ENP type separately. ANOVA analyses were done with SPSS for Windows statistical software (SPSS Inc., Chicago, IL). Post-hoc analysis was performed using a Tukey HSD test on the different concentrations in each soil only if the ANOVA analysis yielded significant results. In the control experiment, a two-way ANOVA was applied using two independent variables; contaminant type and sterilization procedure. Furthermore, two independent t-test analyses were performed between the different contaminants and control within the sterile and non-sterile groups, and between the sterile and non-sterile group but within the same contaminant type. 

## Results

### Microbial community function

**Figure 1:**
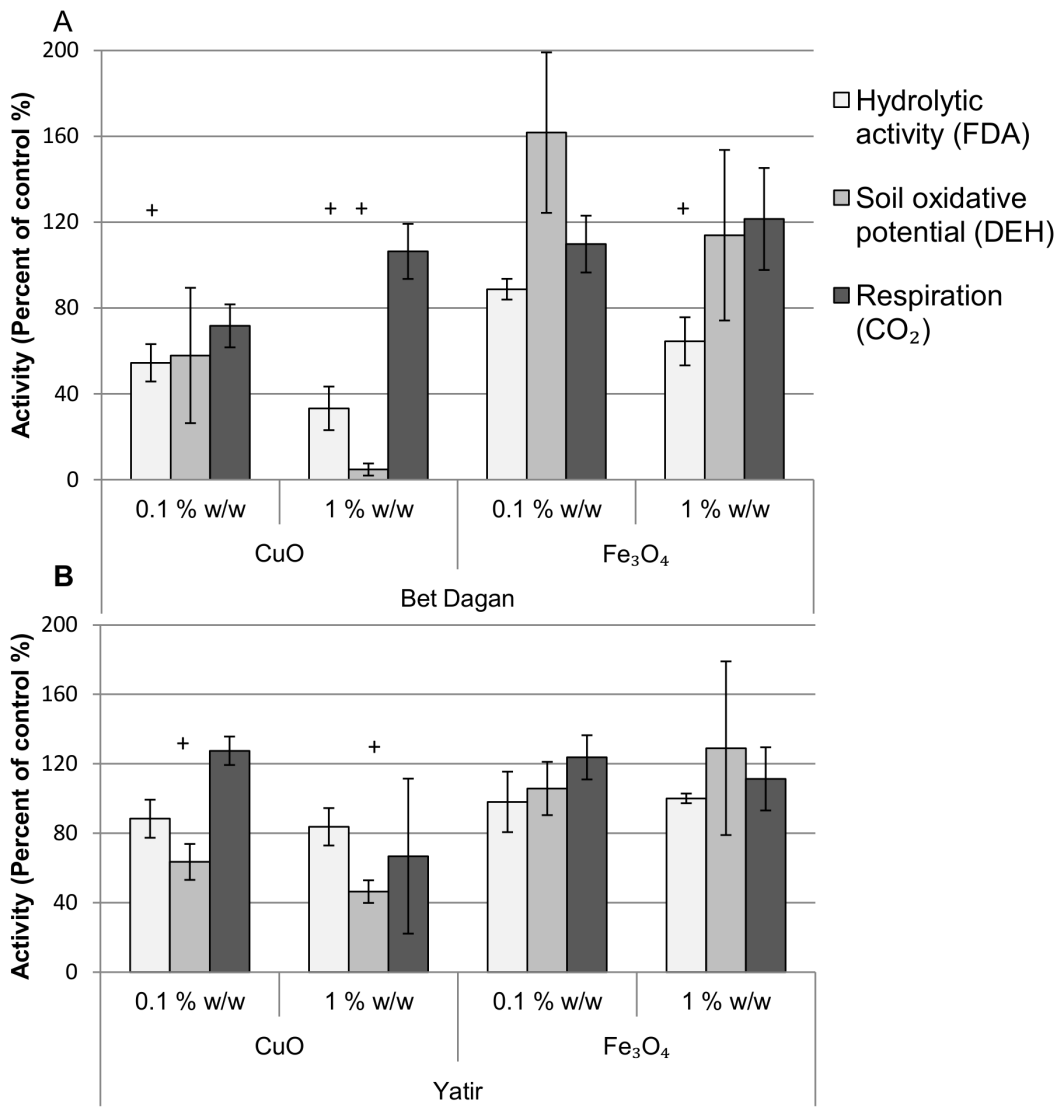
Effect of MO-ENPs on microbial activities in soil. Enzymatic assays of Bet-Dagan (A) and Yatir (B) soil exposed to two MO-ENPs, CuO and Fe_3_O_4_, in two concentrations, 0.1 and 1% w/w. White: FDA assay(n=5); light gray: DEH assay(n=5); dark gray: CO_2_ emission(n=3). All results are described as the percent of activity relative to the average activity of unexposed controls. +, treatments that differ significantly from unexposed control (p<0.05).

The soil microbial communities in the two soil types showed a general trend of decrease in dehydrogenase oxidative potential (DEH) and hydrolytic activities (FDA) following exposure to CuO (Figure 1a and 1b). The most affected samples were those subjected to 1% of CuO in sandy loam (Bet-Dagan) soil (Figure 1a), with a significant (p<0.05) reduction of up to 90% in microbial activity, but the reduction was also significant for the 0.1% CuO samples (p<0.05). Sandy clay loam (Yatir) soil microbial communities showed a reduction in mean activities, although to a lower extent than Bet-Dagan soil samples (Figure 1b); the greatest reduction was only to 50% in DEH activity (p<0.05) and an insignificant decrease in FDA activity relative to the activity in the control soil. Fe_3_O_4_ addition led to a reduction in FDA activity only in Bet-Dagan soil. A mean ~25% increase in DEH activity was measured in Yatir soil samples after the addition of 1% Fe_3_O_4_ with respect to the control (p=0.077). Measurement of ammonia oxidation potential showed similar results to the FDA and DEH assays (data not shown). 

**Figure 2:**
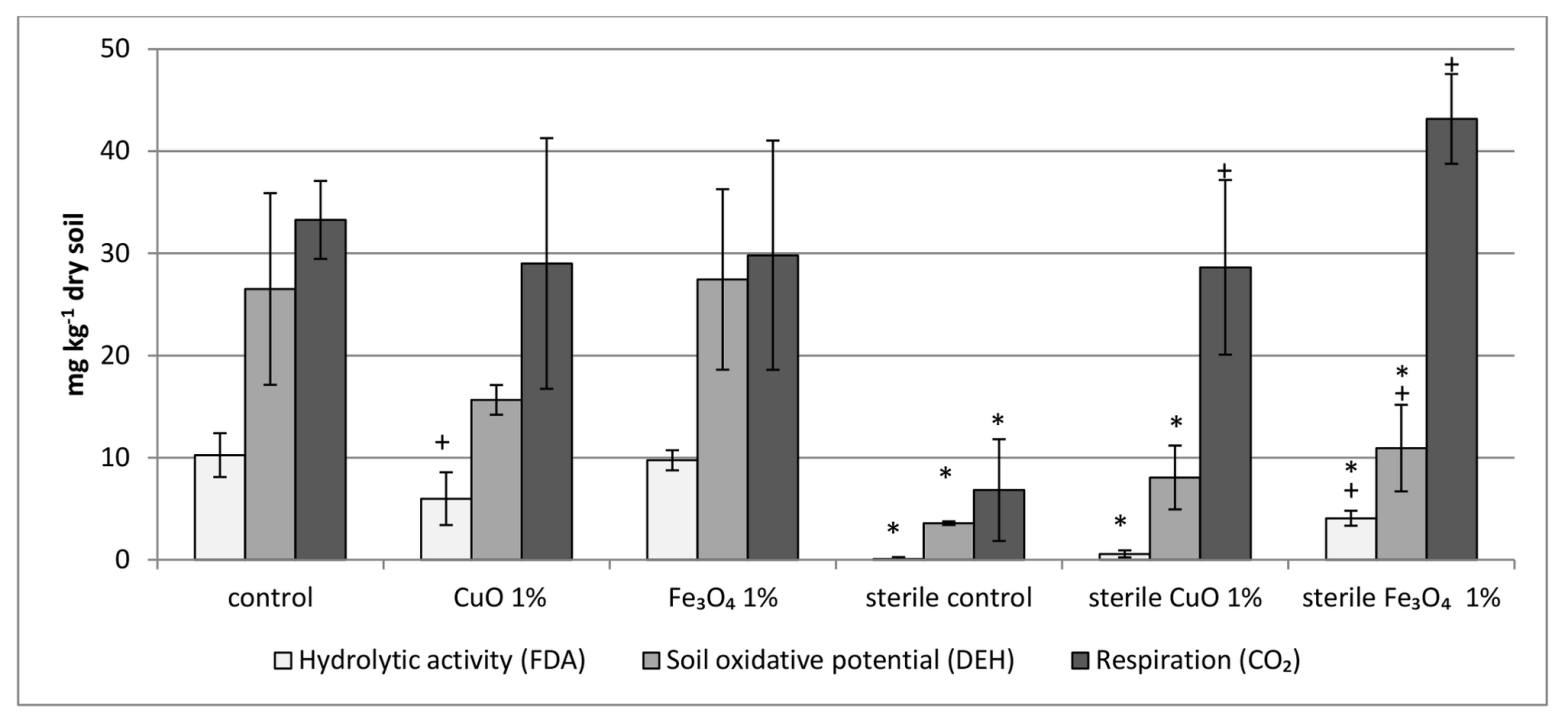
Effect of ENPs on sterilized soil. Soil microbial enzymatic activity measured by three assays FDA, DEH and CO_2_ on sterile and non-sterile Yatir soil for the assessment of non-biological effects of the two ENP types (for all methods and treatments, n=3). +, treatments that differ significantly from unexposed control (p<0.05) and *, treatments that differ significantly from unsterilized controls (p<0.05).

CO_2_ emission (Figure 1a and 1b), an indicator of microbial respiration, was not significantly different between the control and treatments. These results were unexpected because other activities showed different, mostly decreasing, trends. To understand whether these results are methodological artifacts or an authentic biological/chemical phenomenon, a control experiment using sterilized soils was conducted. Examination of sterilized soil subjected to these MO-ENPs suggests the CO_2_ emission to be non-biological, driven by chemical reactions with the MO-ENPs. These results are evident by the amount of CO_2_ evolution measured in sterilized soils with MO-ENPs, compared to their sterilized control MO-ENPs (Figure 2). Furthermore, FDA and DEH enzymatic assay measurements in sterilized soil were also higher when MO-ENPs were present (Figure 2). For example, DEH assay results, indicative of soil oxidative potential, in sterilized soil exposed to Fe_3_O_4_ exceeded by more than 50% the DEH results in unexposed sterilized soil. FDA assay results changed from undetectable in the sterilized control to approximately 5 mg/kg in the Fe_3_O_4_ exposed soil. In this study, the water added to the sterilized soil was not sterilized and therefore modest biological activity was viewed in the sterilized controls without MO-ENPs. The activity in the sterilized control was significantly (p<0.05) lower than in the unsterilized control, showing 80%-100% decrease in all three assays.

### Bacterial community composition and size

**Figure 3:**
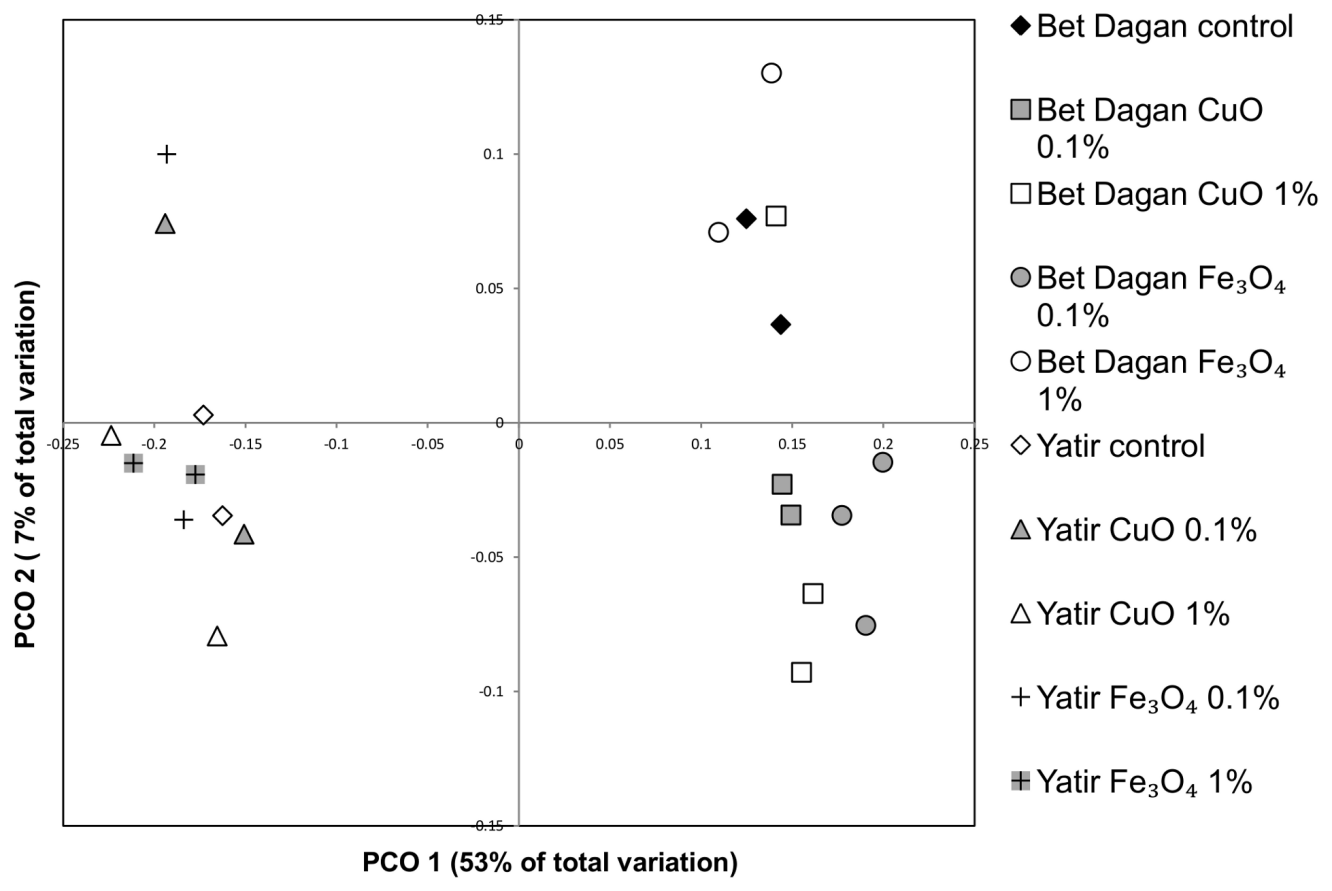
Soil bacterial community similarity of Yatir and Bet-Dagan using 16S rRNA gene sequencing data. PCoA of the Yatir and Bet-Dagan soils with the different ENPs and control, based on UniFrac distance matrix of all samples (n=2 or 3).

The DGGE results showed high consistency with the high-throughput sequencing results and were therefore used as support. Both DGGE fingerprinting and sequencing data indicated that the bacterial communities in the Bet-Dagan and Yatir soils were distinct, regardless of the treatment. This was manifested by low similarity between the bacterial communities of the two soil types, seen in the community fingerprints obtained by DGGE (Figure S1), highly pronounced in the PCoA (Figure 3) ordination of the sequencing data, and confirmed statistically (p<0.05) by AMOVA. Each soil clustered separately on the first factor (contributing 53% of the variance) of the PCoA. 

Analysis of Bet-Dagan soil demonstrated that Fe_3_O_4_, and even more so, CuO application, caused a substantial shift in community composition. PCoA analysis of the sequence data (Figures 3 and S2) revealed that MO-ENP application changed the community composition significantly (p<0.05). Samples of Bet-Dagan soil contaminated with CuO 1% showed high variance in their bacterial community composition. This variance within replicates of the bacterial communities sampled in CuO 1% treatments prevented recognition of a significant difference from the control. However, community composition of this soil contaminated by 0.1% CuO clustered significantly away from the control samples. Despite the little change in activity, community composition of both samples exposed to Fe_3_O_4_ (0.1% and 1%) in Bet-Dagan soil differed significantly from the control community (p<0.05). Samples that were exposed to 0.1% Fe_3_O_4_ showed higher deviation from the control than those exposed to 1% Fe_3_O_4_. Yatir soil bacterial community did not yield significant differences between treatments due to MO-ENPs exposure of both types. Thus, in contrast to the susceptibility to change of the community in Bet-Dagan soil, samples of Yatir soil showed relatively high resistance to change. Despite the overall resistance, 1% CuO did cause some shift in the Yatir community, detected by the DGGE fingerprint (Figure S1b).

Quantification of total bacteria in soil samples, measured by the amount of 16S rRNA gene targets per 1 g of dry soil, as revealed by quantitative PCR (Table 1), showed changes in bacterial population size of Bet-Dagan soil as a result of MO-ENPs exposure but not in Yatir soil. A significant decrease in total bacteria of approximately one fourth of the control community size was found in Bet-Dagan soil samples exposed to 0.1% and 1% CuO (p<0.05). However, only an insignificant decrease in population size can be attributed to the exposure to Fe_3_O_4_ in both concentrations. Furthermore, results showed higher richness values, as well as lower coverage values in Yatir soil, relative to Bet-Dagan soil, in both untreated soils and in MO-ENP exposed soils (Table 1). These results support the data indicating little changes in activity and community composition compared to the control in Yatir soil.

**Table 1:** Diversity indices of the two different soils (Bet-Dagan and Yatir) with the different ENPs CuO and Fe_3_O_4_ as measured by 454 sequencing (n=2 or 3).

Treatments	Observed richness (±SD)	Sampling coverage (±SD)	Bacterial 16S targets (±SD) Per 1 gr soil
Bet-Dagan control	279 (29)^a^	0.77 (0.03)	4.05E6 (7.55E5)
Bet-Dagan CuO 0.1%	277 (12)^a^	0.78 (0.02)	1.47E6 (8.34E5)^*+*^>
Bet-Dagan CuO 1%	242 (18)^a^	0.79 (0.02)	1.06E6 (4.45E5)^*+*^
Bet-Dagan Fe_3_O_4_ 0.1%	237 (22)^a^	0.82 (0.03)	2.36E6 (1.23E6)
Bet-Dagan Fe_3_O_4_ 1%	214 (77)^a^	0.84 (0.08)	4.65E6(4.03E6)
Yatir control	350 (14)^b^	0.69 (0.03)	9E5 (8.45E5)
Yatir CuO 0.1%	362 (21)^b^	0.70 (0.04)	9.7E5 (5.2E5)
Yatir CuO 1%	344 (0)^b^	0.69 (0.001)	4.6E5 (3.37E5)
Yatir Fe_3_O_4_ 0.1%	384 (8)^b^	0.66 (0.001)	4.23E5 (2.37E5)
Yatir Fe_3_O_4_ 1%	365 (3.5)^b^	0.68 (0.01)	2.11E5(2.25E5)

Statistical differences are marked as: samples marked by (a) are significantly different than samples marked by (b). Further, the total bacterial community size as measured by qPCR (n=5): +, treatments that differ significantly from unexposed control (p<0.05).

**Figure 4:**
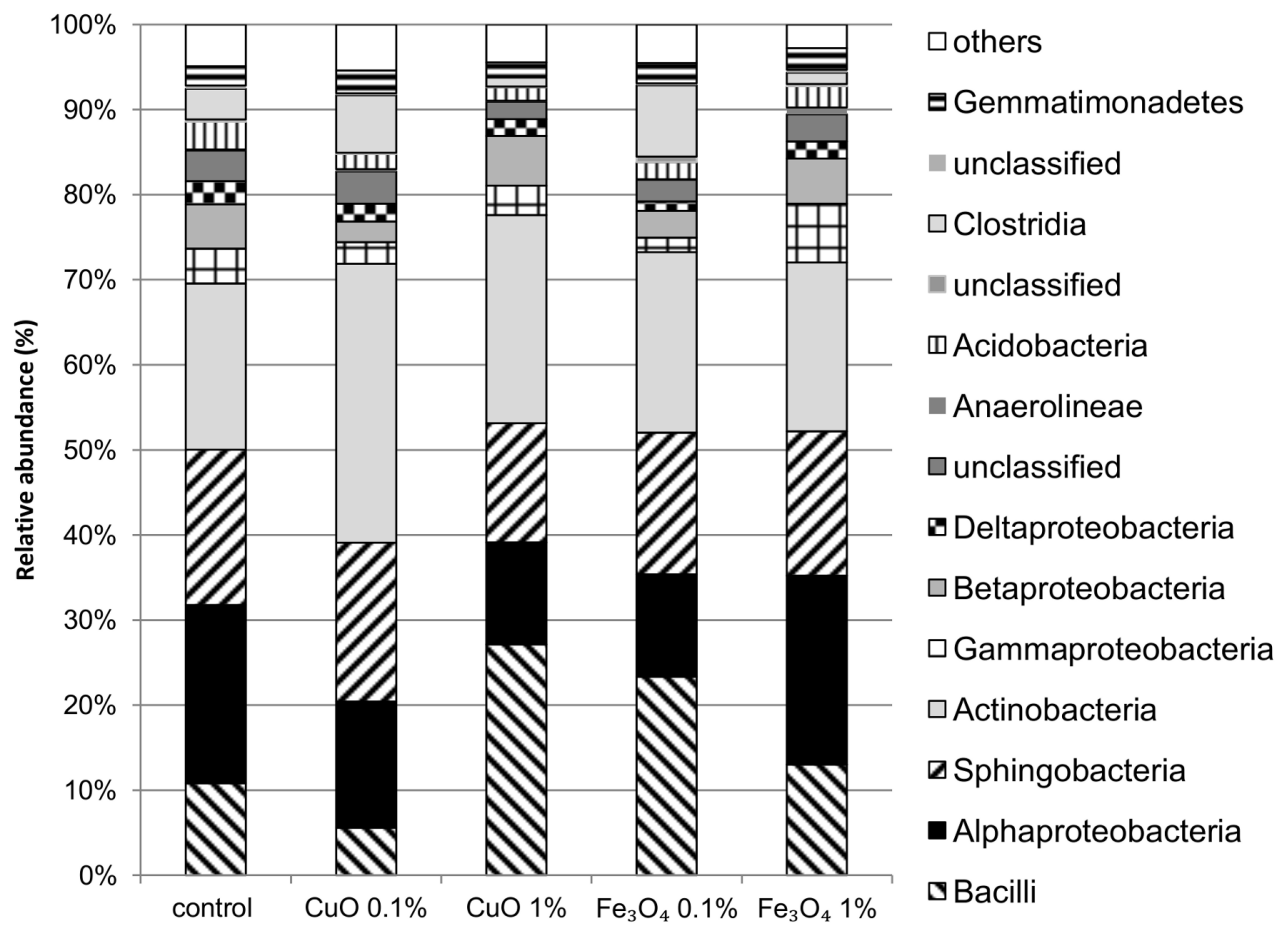
Bet-Dagan soil class level community composition. Composition of bacterial community in Bet-Dagan soil grouped in the class level taxonomy (n=2 or 3).

**Figure 5:**
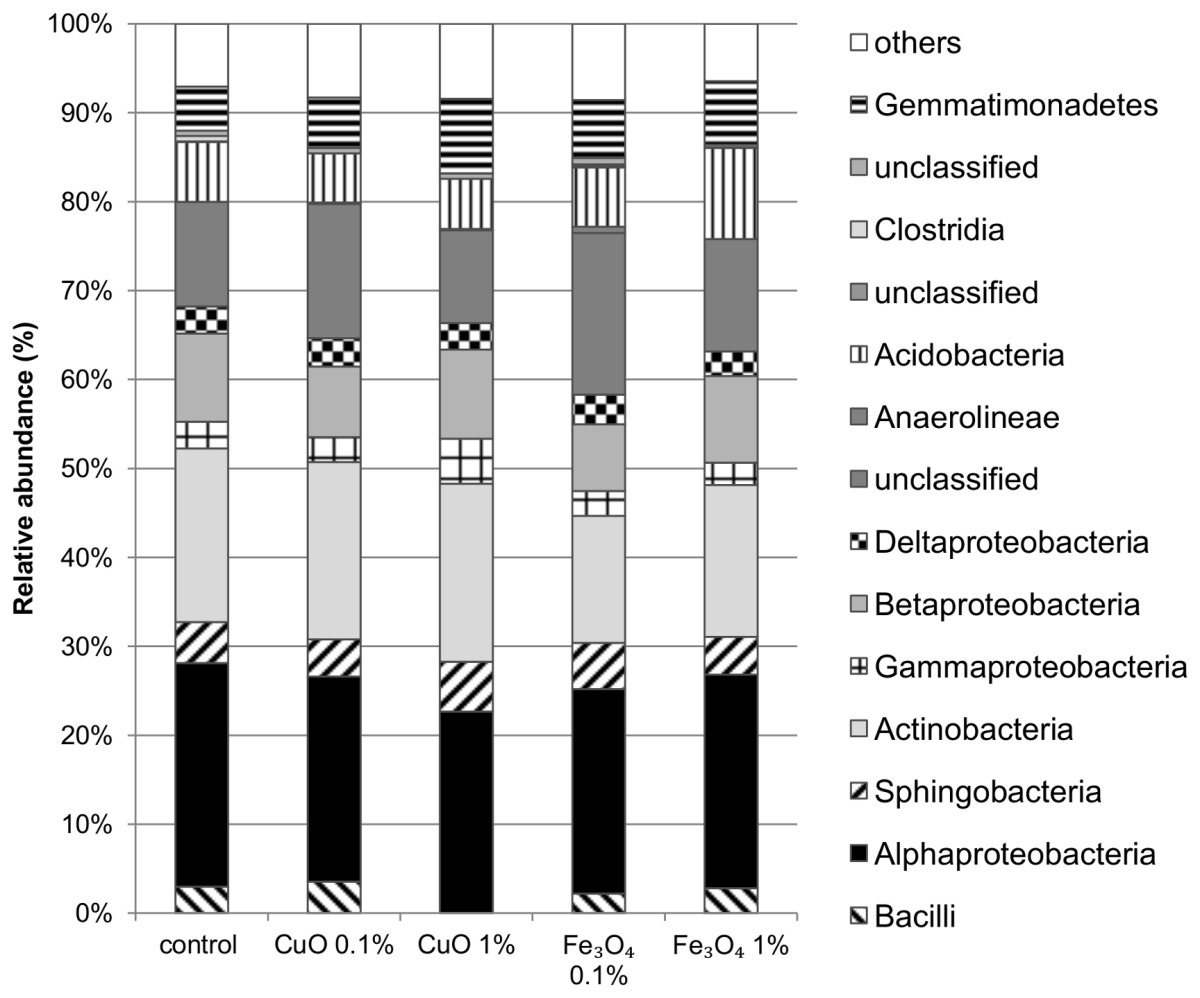
Yatir soil class level community composition. Composition of bacterial community in Yatir soil grouped in the class level taxonomy (n=2).

Phylogenetic distribution of OTUs in Bet-Dagan soil showed significant shifts at the class level community composition due to the different treatments, when compared to the control. A major change caused by the treatments in this soil was an increase in relative abundance of Actinobacteria in soil treated with 0.1% CuO (Figure 4). A decrease in Alphaproteobacteria class members was seen within all treatments of this soil, except in 1% Fe_3_O_4_. Further changes included an increase in abundance of Bacilli class member in treatments CuO 1% and Fe_3_O_4_ 0.1% and an increase in abundance of Betaproteobacteria in CuO 1% treated soils. Unlike Bet-Dagan, Yatir soil did not change significantly in any class abundance except for a decrease in the Bacilli relative abundance in this soil treated with 1% CuO (Figure 5). In order to receive the actual abundance of Bacilli class in soil, their relative abundance was multiplied by the quantitative PCR data. Results suggest that the Bacilli abundance in Bet-Dagan soil exposed to 0.1% CuO decreased to 10% of the control while the total bacterial population decreased to only 20% of the control. On the other hand, in samples exposed to 1% CuO, the Bacilli class abundance decreased to 38% of the control while the total population decreased to 15% of the control population size. In Yatir soil, after exposure to1% CuO, the Bacilli class abundance was eliminated while the total population did not change significantly. 

Metastats results show that in both soil types, specific OTUs reacted to treatments by changing in abundance; some of these OTUs reacted to the treatments differently depending on soil type. Bet-Dagan samples (Figure S3 and Table S1) had many OTUs that showed different abundance in treatments; OTUs 1-*Bacillales* and 584-*Bacillales* showed a significant increase in relative abundance after the addition of 1% CuO and together comprised 20% of the bacterial community in this soil. 1-*Bacillales* showed also a significant decrease after the addition of 0.1% CuO to Bet-Dagan soil and contributed only about 3% of the community. 45-*Chitinophaga*, 43*-Rubrobacter* and 153*-Clostridium* showed high abundance in 0.1% CuO contaminated samples. 3-*Sphingobacteriaceae* and 16- *Sphingomonas* had low abundance in 0.1%, 1% CuO and 0.1% Fe_3_O_4_ Bet-Dagan contaminated samples but significantly higher abundance in 1% Fe_3_O_4_ contaminated samples. 2-*Rhizobiales* abundance was significantly reduced in 1% CuO samples. 11-*Oxalobacteraceae* showed high abundance in 1% CuO contaminated Bet-Dagan soils but was relatively low at 0.1% CuO, compared to the control samples. On the other hand, in the Yatir soil, only three OTUs were affected by CuO 1% treatment; two of them were Bacilli related (1-*Bacillales* and 4-*Bacillus*), which decreased in abundance. The third affected OTU was 35-*Oxalobacteraceae* of the Betaproteobacteria class, which increased in abundance as a result of 1% CuO addition (Figure S4 and table S2).

## Discussion

In the current study, addition of CuO and Fe_3_O_4_ ENPs was found to lower soil microbial activities and change bacterial community composition in Bet-Dagan soil, which is low in organic matter and soil clay fraction. On the other hand, Yatir soil, which has higher organic matter content as well as higher clay fraction, showed only slight changes in the activity and composition of the bacterial community. A recent review [13] discussed and presented different studies that showed MO-ENP reactivity to be dependent on both soil type and nanoparticle size. ENPs in a range under ~10 nm (depending on type) are much more reactive, but as a result could lose reactivity more quickly. For both CuO and Fe_3_O_4_, a mixture of particle sizes (<50 nm) was used in both soil types of this study, leaving the soil type as the only major factor of influence. Soil characteristics such as organic carbon content, grain size fractionation and mineral composition were shown to have a clear, but complex effect on the toxicity of such contaminants [17]. For example, interactions of metallic compounds with clay particles are known for many metallic elements, including Ni, Cd, Co and Pb, and in many cases determine the fate, transport and toxicity of these metals in the environment [47]. These interactions include ENP transformations, such as crystal growth, dissolution, aggregation and aging, which change the micro- or nano-environment surrounding the ENPs [13]. Soils in which the MO-ENPs more readily aggregate could retain MO-ENPs longer, but in a less reactive state. The microbial community in Yatir soil with higher organic matter concentration and larger clay fraction compared to Bet-Dagan soil was also more resistant to the effect of these MO-ENPs, probably due to interactions such as mentioned above. High diversity and richness are good estimators for functional stability in any ecological system, including soil encountering perturbations [48]. Higher richness, as seen in Yatir soil, may support several species holding the same traits (functional redundancy), and in case of disturbance followed by loss of species, there would be minor change in the community activity level. Furthermore, high initial species diversity and richness help soil bacterial communities return to normal function after a disturbance [49]; these parameters further support the relative stability of Yatir soil.

The two soil types had different community compositions, which the MO-ENPs affected differently. Results described high abundance of the class Bacilli in contaminated Bet-Dagan soil, which may indicate this class’ resistance to CuO at concentrations of 1%. The resistance of members of the Bacilli class, namely OTUs 1- *Bacillales* and 584-*Bacillales* to 1% CuO ENPs concurs with results of previous studies showing high abundance of *Lactobacillales* in Cu exposed soil [30] and after exposure to silver NPs [28]. Despite the phylogenetic differences between the *Lactobacillales* and *Bacillales*, they hold one important common characteristic, namely the ability to sporulate. Members of the Bacilli class have been reported to persist in heavy metal polluted soils [50–52] and specifically in copper polluted soils [51]. Copper ion resistance mechanisms are known for many bacterial groups, including gram positive bacteria such as Actinobacteria and Firmicutes [53,54]. Thus, by possessing resistance mechanisms that enable these bacteria to reproduce in the presence of MO-ENPs, together with spore formation abilities that enable them to survive acute exposures, Bacilli may have a considerable advantage at high CuO concentrations. 

Other bacterial populations were affected by the contaminants in this study, including OTUs from *Sphingobacteriaceae, Sphingomonas* and *Rhizobiale* genera. Apart from the report on *Lactobacillales*, Collins et al. [30] also described high sensitivity of *Sphingomonadales* to Cu and ZnO, which is in agreement with the present results. In contrast, they reported that members of the *Rhizobiales* showed low sensitivity to Cu and ZnO, whereas here we observed a decrease in relative abundance of an OTU identified as *Rhizobiales*, as a result of CuO exposure at both concentrations. Despite the consistencies that were found with previous studies, a deeper phylogenetic resolution is needed to determine which genera have specific tolerance or sensitivity to these contaminants. This is due mainly to the fact that each of these bacterial groups - *Rhizobiales*, *Sphingomonadales* and *Bacillales* - include many species that do not react similarly to the discussed compounds. 

The result of this study show that relative abundance of OTUs of the Bacilli class more than doubled in Bet-Dagan soil with 1% CuO, compared to the control, despite the actual lower number of 16S rRNA gene copies measured. Hence, spore formation was the major strategy of survival for the Bacilli members, rather than resistance mechanisms that enable reproduction. Surprisingly, the Bacilli in Yatir samples exposed to 1% CuO and in Bet-Dagan exposed to 0.1% CuO decreased in abundance relative to the total bacterial population. At lower CuO stress levels, as seen after exposure concentrations of 0.1% in Bet-Dagan and 1% in Yatir, there was an increase in abundance of *Oxalobacteraceae*, in Yatir and Bet-Dagan soils, and of *Rubrobacter*, *Clostridium* and *Chitinophaga*, only in Bet-Dagan soil. These groups may possess some resistance mechanisms, possibly other than sporulation, that enable them to survive in the contaminated soils. These groups were also reported to succeed in soils contaminated by metals and heavy metals [54–56]. Also, it is possible that in the low CuO concentration, the Bacilli did not initiate sporulation processes, which may have been a reason for survival in the high CuO concentration. These differences between relative abundance of the discussed populations illustrate the differences between moderate to acute exposure and the role of soil type in mitigating these effects. When an acute dosage of CuO (i.e., 1% for Bet-Dagan) was present in soil, bacteria annotated to Bacilli OTUs increased in relative abundance. This increase was due to a more dramatic decrease in the total bacterial population than that seen for Bacilli. On the other hand, when a similar dosage was given in the Yatir soil, which did not reach acute levels due to soil characteristics, the same OTUs did not have any advantage. 

The two MO-ENPs affected soil microbial activities and bacterial populations differently, with Fe_3_O_4_ having very little negative effect. It is suggested that this low toxicity of Fe_3_O_4_ is partly because it can be found naturally, in both bulk and nanoparticle sizes, in many soil types [4], and is also described as a byproduct in bacterial anaerobic and aerobic metabolism [57,58]. Previous study on the effect of this ENP on soil microbial activities showed an increase in microbial activity as a result of its addition [32]. In the current study, although a reduction in hydrolytic activity was detected as a response to the addition of 1% Fe_3_O_4_ to the Bet-Dagan soil, none of the other activities was affected. 

Chemical reactions were the main cause of increased CO_2_ emission in sterile soil contaminated with ENPs, relative to control soil. Such reactions can include changes to the soil organic matter [17] or changes in mineral adsorption/desorption in soils that could affect carbon dioxide release [13]. The measurements of FDA and DEH enzymatic assay in sterile soil, along with a recent report by Ben-Moshe et al. [17] describing changes in soil organic matter profile when applying CuO and Fe_3_O_4_ ENPs, support the hypothesis that MO-ENPs interacted with soil organic matter. Thus, chemical reactions between the ENPs and the organic matter may have led to high CO_2_ evolution and enzymatic-like activity measurements, even while the actual biological activity was low. These interactions suggest that the negative effect of the MO-ENPs on microbial activity could be higher than was actually measured and previously described, i.e., the actual microbial enzymatic activity in exposed samples was lower than that found in the measurements. It is therefore important for future studies to include a sterile control for assessing non-biological interactions between the MO-ENPs and the soil minerals and organic matter. Soil sterilization using autoclaving changes many soil properties, including pH level and free amino acid content [59,60]; this may, in turn, affect measured MO-ENP reactivity. Because of the bias that the sterilization processes could cause, calculation or normalization of the non-biological effect for our samples was not possible. 

In conclusion, soil type is a key component dictating MO-ENP toxicity to the microbial community it hosts, determining both the bacterial community composition dwelling in the soil and the activity of this community. It is clear from this work that the Yatir soil was more resistant to the applied MO-ENPs, possibly due to the higher organic matter concentrations, clay fraction of the soil and higher native community richness and diversity. The bacterial populations reacting to a MO-ENP, especially CuO, are highly dependent on the concentration of the MO-ENPs applied to soil. We speculate that several different survival strategies are involved in CuO resistance. Low concentrations of CuO (0.1%) could encourage bacteria that are presumably using resistance mechanisms that allow their growth, while in higher concentrations, the relative abundance of spore-forming and resistant bacteria are enhanced due to the decrease in population size of MO-ENP sensitive bacteria. Fe_3_O_4_ has a complex effect on the soil bacterial community, but did not show a straightforward toxic effect on the total soil community. Overall, both MO-ENPs showed an effect on the bacterial community; it is therefore suggested that they should be managed with care and their disposal in the environment should be prevented. 

## Supporting Information

Figure S1 **Effect of MO-ENPs on bacterial community fingerprint in soil.**
(DOCX)

Figure S2 **Bet Dagan soil bacterial community similarity using 16S sequencing data.**
(DOCX)

Figure S3 **Differentially abundant OTUs in Bet Dagan soil.**
(DOCX)

Figure S4 **Differentially abundant OTUs in Yatir soil.**
(DOCX)

Table S1 **The significance of the differentially abundant OTUs in Bet Dagan soil.**
(DOCX)

Table S2 **The significance of the differentially abundant OTUs in Yatir soil.**
(DOCX)
