# Supplementary material for: Effect of Metal Oxide Nanoparticles on Microbial Community Structure and Function in Two Different Soil Types
Source: PLoS One. 2013 Dec 13;8(12):e84441. doi: 10.1371/journal.pone.0084441 (PMC3862805; doi:10.1371/journal.pone.0084441)
Supplement: Figure S1 — Effect of MO-ENPs on bacterial community fingerprint in soil. (DOCX) [file pone.0084441.s001.docx]

Figure S1: Effect of MO-ENPs on bacterial community fingerprint in soil. DGGE fingerprint cluster analysis of the bacterial community in Bet Dagan (A) and Yatir (B) soil based on 16S gene.

**PCR and 16S DGGE fingerprinting methods:**

PCR reactions for denaturing gradient gel electrophoresis (DGGE) analysis were conducted on three replicates for each treatment, as described previously [1]. PCR was performed using a primer set for a general bacterial community: 341F-GC containing a 40 bp GC-clamp to enhance separation in DGGE (clamp sequence in italics), *CGC CCG CCG CGC CCC GCG CCC GTC CCG CCG CCC CCG CCC G*CC TAC GGG AGG CAG CAG [2], and 907R, CCG TCA ATT CMT TTG AGT TT [2]*.* DGGE analysis was performed as described previously [1] with a gradient from 20% to 70% urea and formamide (a 100% denaturant corresponds to 7 M urea and 40% (vol/vol) formamide) in TAE running buffer (2 M Tris base, 1 M glacial acetic acid, 50 mM EDTA). Gels were stained with GelStar Nucleic Acid Stain (0.1 μL/mL of gel solution) (Cambrex Bio Science, Rockland, ME, USA) and photographed using a UV transillumination table (302 nm) with a Kodak digital camera (Rochester, NY). Gel images were subjected to computational analysis using Fingerprinting II software (Bio-Rad laboratories, Hercules, CA) and the Cosine coefficient of the densitometric curve was used as the algorithm for calculating a distance matrix, as previously described [3]. The cluster analysis for three replicates was calculated using an unweighed pair group method with arithmetic means (UPGMA), and with Fingerprinting II software.

1. Green SJ, Michel FC, Hadar Y, Minz D (2004) Similarity of bacterial communities in sawdust- and straw-amended cow manure composts. FEMS Microb Ecol 233: 115–123. Available: http://dx.doi.org/10.1016/j.femsle.2004.01.049.

2. Muyzer G, Hottenträger S, Teske A, Wawer C (1996) Denaturing gradient gel electrophoresis of PCR-amplified 16S rDNA—a new molecular approach to analyse the genetic diversity of mixed microbial communities. Mol microb ecol manual 3: 1–23.

3. Ofek M, Hadar Y, Minz D (2009) Comparison of effects of compost amendment and of single-strain inoculation on root bacterial communities of young cucumber seedlings. Appl Environ Microb 75: 6441–6450.
