# Supplementary material for: Effect of Metal Oxide Nanoparticles on Microbial Community Structure and Function in Two Different Soil Types
Source: PLoS One. 2013 Dec 13;8(12):e84441. doi: 10.1371/journal.pone.0084441 (PMC3862805; doi:10.1371/journal.pone.0084441)
Supplement: Figure S2 — Bet Dagan soil bacterial community similarity using 16S sequencing data. (DOCX) [file pone.0084441.s002.docx]

c

b

a

Figure S2: Bet Dagan soil bacterial community similarity using 16S sequencing data. PCoA with 3 axes, a- 1 & 2, b- 1 & 3 and c-2 & 3, of the Bet Dagan soil with the different ENPs and control; this PCoA is based on a UniFrac distance matrix of only the Bet Dagan samples.
