# Supplementary material for: Effect of Metal Oxide Nanoparticles on Microbial Community Structure and Function in Two Different Soil Types
Source: PLoS One. 2013 Dec 13;8(12):e84441. doi: 10.1371/journal.pone.0084441 (PMC3862805; doi:10.1371/journal.pone.0084441)
Supplement: Figure S3 — Differentially abundant OTUs in Bet Dagan soil. (DOCX) [file pone.0084441.s003.docx]

Figure S3: Differentially abundant OTUs in Bet Dagan soil. The description of treatments that are significantly different (p<0.05) in the different OTUs are presented in Table S1.
