# Supplementary material for: Effect of Metal Oxide Nanoparticles on Microbial Community Structure and Function in Two Different Soil Types
Source: PLoS One. 2013 Dec 13;8(12):e84441. doi: 10.1371/journal.pone.0084441 (PMC3862805; doi:10.1371/journal.pone.0084441)
Supplement: Figure S4 — Differentially abundant OTUs in Yatir soil. (DOCX) [file pone.0084441.s004.docx]

Figure S4: Differentially abundant OTUs in Yatir soil. The description of treatments that are significantly different (p<0.05) in the different OTUs are presented in Table S2 (Cu: CuO; Fe: Fe_3_O_4_).
