# Supplementary material for: Effect of Metal Oxide Nanoparticles on Microbial Community Structure and Function in Two Different Soil Types
Source: PLoS One. 2013 Dec 13;8(12):e84441. doi: 10.1371/journal.pone.0084441 (PMC3862805; doi:10.1371/journal.pone.0084441)
Supplement: Table S1 — The significance of the differentially abundant OTUs in Bet Dagan soil. (DOCX) [file pone.0084441.s005.docx]

Table S1: The significance of the differentially abundant OTUs in Bet Dagan soil. The OTUs (label numbers as in Figure S1) appearing in the table are those that are significantly different among the treatments. These are the results of the “Metastats” function which determines whether OTUs are differentially represented between the treatments.

| Significance p<0.05 | control | CuO 0.1% | CuO 1% | Fe_3_O_4_ 0.1% |
| --- | --- | --- | --- | --- |
| CuO 0.1% | 1, 28, 153 |  |  |  |
| CuO 1% | 1, 2, 584 | 1, 11, 28, 35, 43, 153, 584 |  |  |
| Fe_3_O_4_0.1% | 1, 3, 4 | 1, 4, 18, 28, 40 | 1, 4, 11, 18, 40, 48, 82, 153, 584 |  |
| Fe_3_O_4_1% | - | 1, 3, 4, 7, 14, 28, 43, 45, 153 | 1, 3, 6, 7, 9, 14, 16, 126, 584 | 1, 3, 6, 7, 9, 14, 16, 18, 126 |
